# Artificial Intelligence, Healthcare, Clinical Genomics, and Pharmacogenomics Approaches in Precision Medicine

**DOI:** 10.3389/fgene.2022.929736

**Published:** 2022-07-06

**Authors:** Habiba Abdelhalim, Asude Berber, Mudassir Lodi, Rihi Jain, Achuth Nair, Anirudh Pappu, Kush Patel, Vignesh Venkat, Cynthia Venkatesan, Raghu Wable, Matthew Dinatale, Allyson Fu, Vikram Iyer, Ishan Kalove, Marc Kleyman, Joseph Koutsoutis, David Menna, Mayank Paliwal, Nishi Patel, Thirth Patel, Zara Rafique, Rothela Samadi, Roshan Varadhan, Shreyas Bolla, Sreya Vadapalli, Zeeshan Ahmed

**Affiliations:** ^1^ Rutgers Institute for Health, Health Care Policy and Aging Research, Rutgers University, New Brunswick, NJ, United States; ^2^ Department of Medicine, Rutgers Robert Wood Johnson Medical School, Rutgers Biomedical and Health Sciences, New Brunswick, NJ, United States

**Keywords:** artificial intelligence, healthcare, clinical-genomics, pharmacogenomics, precision medicine

## Abstract

Precision medicine has greatly aided in improving health outcomes using earlier diagnosis and better prognosis for chronic diseases. It makes use of clinical data associated with the patient as well as their multi-omics/genomic data to reach a conclusion regarding how a physician should proceed with a specific treatment. Compared to the symptom-driven approach in medicine, precision medicine considers the critical fact that all patients do not react to the same treatment or medication in the same way. When considering the intersection of traditionally distinct arenas of medicine, that is, artificial intelligence, healthcare, clinical genomics, and pharmacogenomics—what ties them together is their impact on the development of precision medicine as a field and how they each contribute to patient-specific, rather than symptom-specific patient outcomes. This study discusses the impact and integration of these different fields in the scope of precision medicine and how they can be used in preventing and predicting acute or chronic diseases. Additionally, this study also discusses the advantages as well as the current challenges associated with artificial intelligence, healthcare, clinical genomics, and pharmacogenomics.

## Introduction

Precision medicine is the utilization of healthcare tools to create specialized treatments that consist of optimal actions for the patient, based on the data available (König et al., 2017; Pinho, 2017; Gameiro et al., 2018; Ginsburg and Phillips, 2018; Ahmed et al., 2020a; Ahmed, 2020; Elemento, 2020; Faulkner et al., 2020; Ahmed et al., 2021a). As clinical, genomic, and metabolic data become easier to obtain and interpret in relation to complex and chronic diseases such as cancer, disease treatment will become more effective (McAlister et al., 2017; Pinho, 2017; Ginsburg and Phillips, 2018; Goetz and Schork, 2018; Bilkey et al., 2019; Ahmed et al., 2020a; Ahmed, 2020; Faulkner et al., 2020; Ahmed et al., 2021a). In the current state of healthcare, healthcare professionals tend to divide their attention and plan treatments based on symptoms (McAlister et al., 2017; Bilkey et al., 2019). However, symptoms like pain, vary from patient-to-patient and may even be absent in life-threatening situations (Lazaridis et al., 2014; McAlister et al., 2017; Pinho, 2017; Goetz and Schork, 2018; Bilkey et al., 2019). Since symptoms can greatly vary between patients, utilizing genomic and metabolic data in conjunction with clinical data from previous patients enables clinicians can prescribe a better, more personalized treatment plan (McAlister et al., 2017; Goetz and Schork, 2018). Thus, the development and implementation of precision medicine should improve the quality of healthcare compared to the conventional system dominated by symptom-driven medicine (Lazaridis et al., 2014; McAlister et al., 2017; Pinho, 2017; Ginsburg and Phillips, 2018; Goetz and Schork, 2018; Bilkey et al., 2019; Ahmed et al., 2020a; Faulkner et al., 2020; Ahmed et al., 2021a).

International interest in precision medicine could be seen as early as 2011, with the American Association for Cancer Research’s (AARC) Project GENIE, which utilized several “big data initiatives” such as Genomics Evidence Neoplasia Information Exchange (GENIE) (Sweeney et al., 2017). The aim of this project was to address the challenges that came with sharing large amounts of genomics and clinical data, specifically regarding effective cancer therapies (Micheel et al., 2018). This level of innovation in precision medicine coincided with a decline in costs of DNA-sequencing techniques and a more widespread adoption of electronic medical records, which conveniently allowed for sharing and analysis of data (Bentley, 2006; Shendure et al., 2008; Evans, 2016; Kruse et al., 2016; Garrido-Cardenas et al., 2017; Graber et al., 2017; Howe et al., 2018). One common application of precision medicine in the United States is genetic screening, which is used to predict and diagnose critical conditions, which can reduce rates of morbidity (Smed et al., 2021). Another emerging application is the prescription of drugs based on genetic markers of efficacy. For instance, studies have shown that seizure drug carbamazepine having the HLA-B*1502 gene is highly likely to experience adverse effects. However, the integration of these genetic markers in prescribing drugs requires strong evidence of clinical validity first. However, the integration of these genetic markers in the prescription of drugs first require strong evidence of clinical validity at all stages of the drug product cycle (Sweeney et al., 2017; Ginsburg and Phillips, 2018). Despite pharmacogenomics being in the early stages of development, it shows great promise toward driving patient-specific outcomes.

**FIGURE 1:**
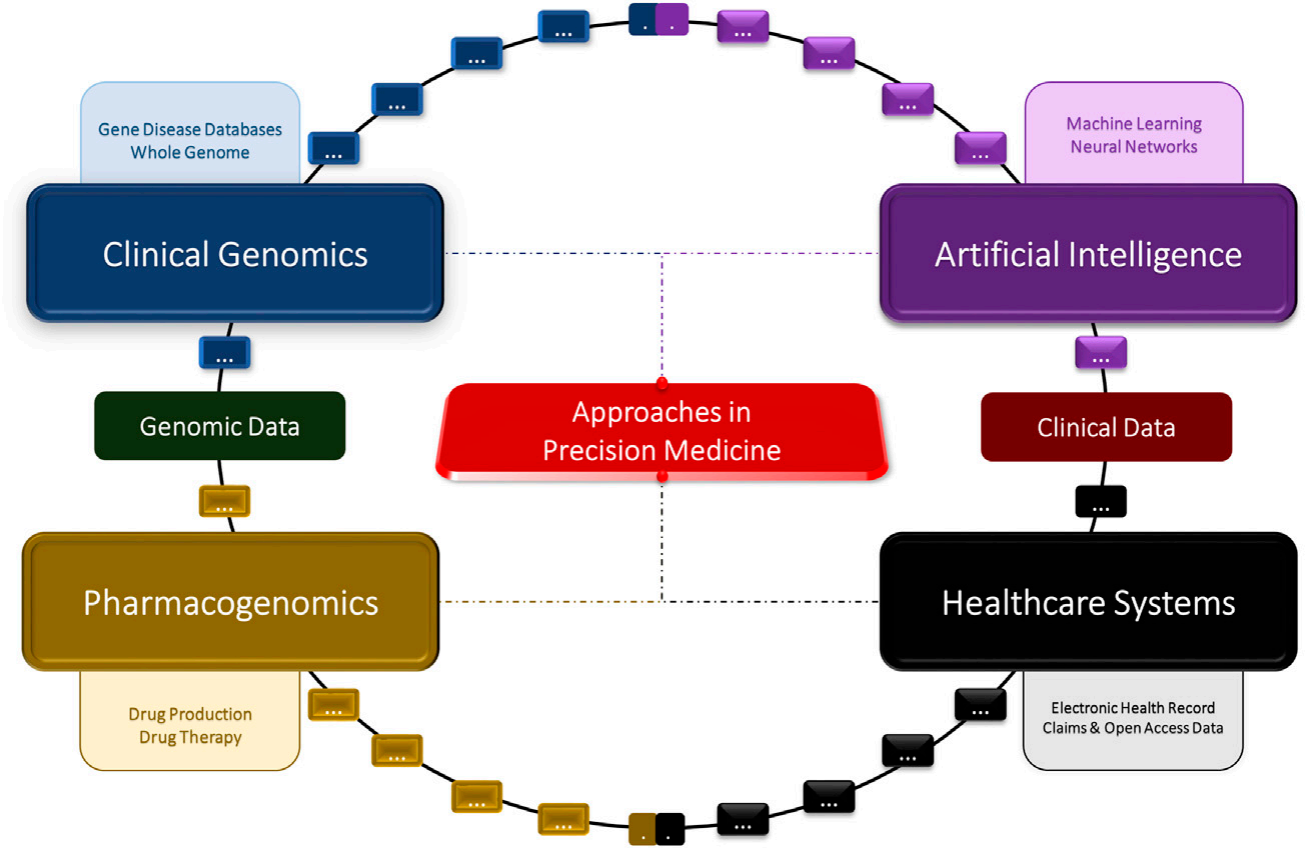
Concept diagram of the artificial intelligence, clinical genomics, pharmacogenomics, and big data approaches in precision medicine.

Artificial intelligence (AI) is a general term used to describe the process of using computers and technology to create stimulating software that resembles human-like critical thinking (Ramesh et al., 2004; Amisha Malik et al., 2019). AI is known to utilize many techniques (fuzzy expert system and artificial neural networks, *etc.*) that can be useful when applied to healthcare (Ramesh et al., 2004; Hessler and Baringhaus, 2018; Amisha Malik et al., 2019; Mintz and Brodie, 2019; Hashimoto et al., 2020). AI can be applied to medicine in two different ways: virtually and physically. The uses of the virtual aspect of AI can range from electronic healthcare records (Esteva et al., 2019) to neural networks guiding patient treatments (McDonnell et al., 2021). The physical subunit of AI involves physical machines like robots assisting in surgeries (Bhandari et al., 2020), or AI-generated prosthetics for the disabled (Bernauer et al., 2021). In addition, ML, or machine learning, is a subunit of artificial intelligence, which utilizes algorithms and code in order to provide personalized experiences, where predictions are backed by mathematical data points (Deo, 2015; Currie et al., 2019). Classical machine learning is heavily dependent on human intervention; however, more unsupervised techniques have been employed in the healthcare field in more recent years (Baştanlar and Özuysal, 2014; Shen et al., 2021). This review focuses on the development of four major fields: AI, healthcare, clinical genomics, and pharmacogenomics, and their overall impacts on precision medicine (Figure 1).

## Healthcare Systems

A healthcare system is the combination of institutions, people, and resources that are involved in delivering health services and care to individuals (Kim et al., 2017; Ahmed et al., 2019a; Ho et al., 2020). The two main types of healthcare systems are commercial and academic (Kim et al., 2017; Ahmed et al., 2019a). Commercial healthcare systems (e.g., eClinicalWorks, praxis, and Allscripts) are characterized by continuous data flow and are utilized by clinical staff to implement patient treatment; therefore, no mistakes can be made with these data because this can lead to a detrimental consequence on humans (Kim et al., 2017; Ahmed et al., 2019a). One example of a commercial healthcare system is EPIC, a popular electronic medical record system, which is used by 45 percent of the United States population to store their records (Epic Systems, 2019; Shull, 2019). Their physicians then utilize the software to monitor their patients’ healthcare from start to finish (Epic Systems, 2019; Shull, 2019). Academic healthcare systems are research based and can be characterized by limited clinical data management and periodic data flow with the overall goal of improving patient treatment (Rim et al., 2016; Kim et al., 2017; Ahmed et al., 2019a). An electronic healthcare record (EHR) is the collection of patient health information that is digitally stored (Knaup et al., 2007; Wronikowska et al., 2021). The compilation of patient information serves to also create an overall understanding of a given population’s health status, for example, the frequency of specific diseases within the population or a specific sub-group (Knaup et al., 2007; Yeh et al., 2020). This information can then be shared across different healthcare settings, and such communication is important toward specific treatment for individuals (Koppel and Lehmann, 2014). Furthermore, an Observational Medical Outcomes Partnership (OMOP) model can be used alongside the electronic healthcare system to efficiently provide data to institutions (Gini et al., 2016; Michael et al., 2020). It was initially launched with the vision of using the system to determine the best practices by using healthcare data and helping patients (Gini et al., 2016; Al-Hanawi et al., 2021).

EHRs can assist healthcare providers in efficiently diagnosing specific rare medical cases that may not be encountered often (Knaup et al., 2007; Yeh et al., 2020; Wronikowska et al., 2021). In recent years, it has become more difficult to improve the health outcomes for a patient by a drastic margin without raising their out-of-pocket costs (Al-Hanawi et al., 2021), thus making it difficult for timely progress toward better patient care (Pastorino et al., 2019; Al-Hanawi et al., 2021). However, these obstacles can be overcome by using Big Data, which relies on its ability to recognize patterns and convert extremely high volumes of data into usable knowledge in the field of precision medicine (Ahmed et al., 2019a; Cammarota et al., 2020; Seyed Tabib et al., 2020). This data can be analyzed in a high-performance computing (HPC) environment where highly intensive computing experiments can be performed (Castrignanò et al., 2020). EHR plays a significant role, as it allows for the communication of patient data across different platforms (Knaup et al., 2007; Yeh et al., 2020), thus maximizing the chance of an effective treatment being developed (Pastorino et al., 2019; Seyed Tabib et al., 2020; Al-Hanawi et al., 2021).

Claims data refers to health insurance claims, which can contain a large variety of information about a specific patient, including details about patient diagnoses, treatments required, and finances (Stein et al., 2014; Moscatelli et al., 2018; Prosperi et al., 2018). In addition, claims data reduce selection bias by giving physicians an overall view of the patient’s continued use of the healthcare system (Stein et al., 2014). These features make claims data desirable in the field of precision medicine (Prosperi et al., 2018). This is due to the availability of a larger subset of data, and the greater the number of individuals the data is pulled from, the more likely it is for a claim made based on that data to be accurate. Complex, multivariable modeling is also important because much of precision medicine is based on the fact that there are many different factors/determinants of an individual’s health (Moscatelli et al., 2018). Clinical data are the compilation of patient data, scattered in numerous databases throughout the healthcare system (McGinnis et al., 2011). Such data often address and assess genetic and metabolomic considerations, individual health, health behaviors, such as lifestyle, environmental factors, and healthcare financing, defines as the patient’s management of funds toward the medical area. Clinical data also address the responses and medical procedures taken toward the health concern (McGinnis et al., 2011; Bram et al., 2015).

## Genomics

Genomics is a subfield of molecular biology concerned with mapping structure and function genomes. The differences between individual genomes are due to the unique biological DNA they are composed of (Woese, 1969; International Human Genome Sequencing Consortium, 2001; Venter et al., 2001; Schneider et al., 2017; Roth, 2019; Zeeshan et al., 2020). Humans have between 20,000 and 25,000 genes, with each gene consisting of between a few hundred to 2 million DNA bases (Woese, 1969; International Human Genome Sequencing Consortium, 2001; Schneider et al., 2017). With the completion of the mapping of the human genome by the Human Genome Project in 2003 (Collins et al., 1998), many new and exciting applications of genomics in the medical field have been made possible. One of these applications can be seen in pharmacogenomics, which allows specialists to assign medication and corresponding dosage based on the patient’s genetic markers (Wake et al., 2019; Cecchin and Stocco, 2020). Another application can be seen in clustered regularly interspaced short palindromic repeats (CRISPR), which allows for efficient gene modification in a variety of organisms. CRISPR-Cas9 has aided in understanding of the disease process through establishing genetically variable disease models (Ma et al., 2014; Cho et al., 2018; Manghwar et al., 2019). Advancements in using CRIPSR-Cas9 has made it possible to potentially treat chronic diseases such as cancers (Chen et al., 2019), leukemia (Tzelepis et al., 2016), HIV (Xiao et al., 2019), β-thalassemia (Frangoul et al., 2021), and sickle cell anemia (Demirci et al., 2019; Frangoul et al., 2021).

### Constituents of the Genome

The phenotypic display of humans is dependent upon the expression of genes, which is affected by numerous factors (Woese, 1969; International Human Genome Sequencing Consortium, 2001; Venter et al., 2001; Schneider et al., 2017; Roth, 2019; Zeeshan et al., 2020). The bridge between genotype and phenotype is proteins, and gene expressions are broken down into 2 stages: transcription (De Klerk and ‘t Hoen, 2015; Polychronopoulos et al., 2017; Dieci et al., 2013) and translation (Simpson et al., 2020; Lyu et al., 2021). RNA splicing, which takes place between the transcription and translation processes, results in large portions of the RNA molecule being removed, with the remaining strands being reconnected (Montes et al., 2019; Zhao, 2019; Wang and Aifantis, 2020; Xu et al., 2021). The sequences that are cut out are known as introns, non-coding intervening sequences within the primary transcript, while the other strands are known as exons, sequences within a primary transcript that remain after RNA processing (Montes et al., 2019; Xu et al., 2021). Due to the presence of introns, a single gene can encode for more than one kind of polypeptide depending on which sequences are treated as exons (Montes et al., 2019; Ule and Blencowe, 2019; Zhao, 2019; Wang and Aifantis, 2020; Xu et al., 2021; Xu et al., 2021). This process is commonly known as alternative RNA splicing and leads to increased diversity in protein coding (Ule and Blencowe, 2019; Zhao, 2019; Xu et al., 2021). However, it is not extensively regulated, and mis-splicing or mutations can lead to diseases such as muscular dystrophy (Takeshima et al., 2010; Fletcher et al., 2013; Scotti and Swanson, 2016) and premature aging dystrophy (Niblock and Gallo, 2012; Scotti and Swanson, 2016). Protein-coding DNA accounts for only 1.5% of the human genome. However, 75% of the genome is transcribed at some point, indicating that a significant amount of the genome is transcribed into non-protein-coding RNAs (ncRNAs) (Mattick and Makunin, 2006; Mishra et al., 2016; Slack and Chinnaiyan, 2019). Non-coding DNA including introns can play a role in the regulation of gene expression such as transcription initiation and termination (Hombach and Kretz, 2016; Montes et al., 2019; Wang and Aifantis, 2020; Xu et al., 2021; Lyu et al., 2021). In addition, some non-coding DNA is transcribed into function non-coding RNA molecules like tRNA (Phizicky and Hopper, 2010), rRNA (Yan et al., 2019), and regulatory RNAs which help with the processes of transcription and translation (Panni et al., 2020). According to RefSeq (O'Leary et al., 2016), a database run by the NCBI, there are currently 20,203 protein-coding genes and 17,871 non-coding genes (Polychronopoulos et al., 2017).

### Next-Generation Sequencing

Next-generation sequencing (NGS) refers to the genome sequencing technologies that began to rapidly develop in the early 2000s (Sanger et al., 1977). Prior to this, Sanger sequencing, a type of sequencing created in 1975, was the primary technique used to sequence DNA (Sanger et al., 1977). With the later development of polymerase chain reaction (Green and Sambrook, 2018) in addition to automated DNA sequencing using fluorescent tags (Metzker et al., 1994; Welch and Burgess, 1999), DNA sequencing became powerful enough to create the first draft of the human genome in 2001 (International Human Genome Sequencing Consortium, 2001; Venter et al., 2001). Currently, Illumina sequencing is the most popular sequencing technology due to its accuracy, cost, and speed (Liu et al., 2012). Illumina sequencing belongs to a family of NGS technology that produces short reads (50–300 base pairs), with the most notable other technology in this category being Ion Torrent sequencing (Rhoads and Au, 2015; De Coster and Van Broeckhoven, 2019). Long-read sequencing technologies created by Oxford Nanopore (Green and Sambrook, 2018) and Pacific Biosciences (Rhoads and Au, 2015) generate reads that are thousands of base pairs long, but their reads are generally lower quality than those created by short-read sequencing (Rhoads and Au, 2015; Jain et al., 2016; Lahens et al., 2017). However, due to great interest in structural variants (genomic alterations that can be thousands of base pairs long) and their effects on diseases, improvements need to be made to long-read sequencing to avoid the costs that short-read sequencing reads suffer from such as low sensitivity (30–70%) when detecting structural variants (Liu et al., 2012; De Coster and Van Broeckhoven, 2019; Levy and Boone, 2019). After sequencing data is collected, the FASTQ file format is the traditional format used to share this information. FASTQ is a type of plain text files formatted such that each sequence has four corresponding lines of text. These lines contain information such as the sequence identifier, nucleotide sequence, a “+” sign to indicate the end of the sequence, and a line of quality values corresponding to the sequence of bases recorded in the second line (Cock et al., 2010). For storing the reference genome, FASTA, another text-based file format, is commonly used. Using the reads from the sequencing and the reference genome, algorithms map the reads to the reference genome, and these results are stored in either a Sequence Alignment Map (SAM) or its binary equivalent (BAM) file (Li et al., 2009; Hoogstrate et al., 2021). A SAM file is readable by humans, but since file sizes are so large, BAM files are used to compress the data (Yohe and Thyagarajan, 2017). Finally, since genetic variation is of interest in research, variant call format (VCF) files are commonly generated based on this data, which are files that describe the sequence variations, insertions, and deletions found in the samples along with rich annotations (Zhang, 2016; Morash et al., 2018).

NGS data can supplement other genomic sequencing methods and further develop the capabilities of DNA sequencing, which can significantly improve the effectiveness of precision medicine (Yohe and Thyagarajan, 2017; Morash et al., 2018). In conjunction with whole-genome sequencing (WGS), which has become more affordable, NGS can improve disease risk detection with improved methods for analyzing sequenced data. This assists in the development of precision medicine (Yohe and Thyagarajan, 2017; Zeeshan et al., 2020; Caspar et al., 2021). Reporting on NGS utilization, several studies have shown its efficiency in identifying actionable genetic mutations in cancer patients, developing drugs to target tumors, and utilizing sequencing results to match patients to therapy methods (Yohe and Thyagarajan, 2017; Morash et al., 2018; Nakagawa and Fujita, 2018; Morganti et al., 2019; Zhao et al., 2019; Caspar et al., 2021). While the current effectiveness of NGS data in precision medicine remains controversial, primarily due to the experimental design of studies determining the efficacy of NGS data, and there is still immense potential for development (Yohe and Thyagarajan, 2017; Nakagawa and Fujita, 2018; Morganti et al., 2019).

### Variant Discovery

WGS and WES are two different types of NGS, which are more efficient and accurate methods of DNA sequencing than traditional Sanger sequencing (Ahmed et al., 2019b). These two techniques can be used to find different variants in an individual’s DNA that may be of clinical significance (may relate to the appearance of a specific disease). WGS involves the sequencing of an individual’s entire genome, including both exons (protein-coding regions) and introns (non-protein coding regions) (Meienberg et al., 2016; Petersen et al., 2017). WES, however, only involves the sequencing of exons, or protein-coding regions (Petersen et al., 2017; Ahmed et al., 2019b). Variants in the human genome are changes in the DNA/nucleotide sequence that may or may not result in changes to the protein-encoding transcript and protein-building process as a whole (Petersen et al., 2017). There are several different types of variants that can be detected by the WGS and WES processes, such as single-nucleotide polymorphisms (SNP) and short or long insertions or deletions polymorphism (indels) (Petersen et al., 2017; Ahmed et al., 2021b). Variant calling or the identification of different types of variants is important because it can help researchers develop a greater understanding of the genetic components of various diseases, which can aid in future diagnoses and disease prevention (Ahmed et al., 2021b). There are three main types of WGS and WES pipelines: cloud-computing, centralized, and standalone (Ahmed et al., 2021b). The main differences between these three pipelines are the environments in which they are applied. Cloud-computing pipelines are used in environments with, “on-demand compute resources managed and provided by external vendors” (Petersen et al., 2017; Ahmed et al., 2021b). Centralized pipelines are used in local computers (Petersen et al., 2017). Standalone pipelines are mainly used in high-performance computing environments (Petersen et al., 2017). These pipelines are designed to effectively collect and process the data from WGS or WES in a way so that it can be used by researchers or medical professionals to best recognize the links between genetic variants and diseases (Petersen et al., 2017; Ahmed et al., 2021b; Ahmed et al., 2021c).

## Clinical Genomics

The gene–disease relationship involves the detection of diseases associated with numerous genes with the help of sequencing techniques (Ahmed et al., 2020b). Applications can be used to help organize and assimilate information from the genomic data with the phenotypic data. By doing so, the gene–disease relationship can improve precision while detecting abnormalities in patients. They can also predict patient susceptibility to a particular disease and open the possibility of treatment options of rare diseases (Strande et al., 2017; Hu et al., 2021; Megías-Vericat et al., 2021). The study of this association can also help elucidate gene function (Van Dam et al., 2018), estimate the prevalence of genes in populations (Zhou and Skolnick, 2016), differentiate among subtypes of diseases (Nakatsuka et al., 2017) and trace how genes may predispose to (Sørlie et al., 2003) or protect against illnesses (Pirmohamed, 2006), and improve medical intervention (Ahmed et al., 2020b; Wickenhagen et al., 2021).

Gene–disease databases are essential due to their unique display of information regarding the “exchange and reporting of actionable genetic variants and associated phenotype” (Ahmed et al., 2020b). This information is vital because it can be used to aid in treatment for complex diseases like cancer, where detection of just a few vital variants can help warn about the development of a tumor (Orkin and Bauer, 2019). An example of gene–disease databases is a database of disease–gene associations with annotated relationships among genes (eDGAR), which collects and arranges data related to gene/disease associations along with interactions in heterogenous and polygenic diseases (Huang et al., 2018). With a focus on “the structural and functional annotations of the genes” (Huang et al., 2018), the database provides the cytogenetic location of a gene. When multiple sets of genes are prevalent in the same disease, the data are organized in such a way that it allows for customized data search (Huang et al., 2018). This is important because it allows for multiplatform usage and comparability so users can get the information that they need as accurately as possible in order to tailor procedures specific to their patients or research (Huang et al., 2018).

As the technology for genomic analysis advances, more reports of gene–disease relationships are beginning to expand, facilitating the need for accessibility to information storage (Babbi et al., 2017; Ahmed et al., 2020b). The ability to analyze a database containing information about genetic diseases and cross referencing that data with patient records has the potential to treat a genetic disease before it proliferates (Babbi et al., 2017; Huang et al., 2018). There are approximately 18,000 gene–disease databases that collect data (Huang et al., 2018). Out of these 18,000, there are approximately 50 authentic databases. However, there are no existing databases that cover the entire human genome. Examples of these gene–disease databases are Ensembl (Landrum et al., 2016), GenCode (Aken et al., 2016), ClinVar (Frankish et al., 2019), GeneCards (Landrum et al., 2016), HGMD (Stelzer et al., 2016), OMIM (Stenson et al., 2012), Orphanet (Amberger et al., 2015), SwissProt (INSERM, 1997), and LncRNADisease (Consortium, 2015). Table 1 further details the extensive availability of various databases that contain raw and multi-omics data. Very few of these gene–disease databases are approved by the American College of Medical Genetic and Genomics (ACMG), a medical organization that focuses on improving health through medical genetics and genomics.

**TABLE 1:** Multi-omics/genomics databases: Ensembl, GENCODE, ClinVar, GeneCards, HGMD, OMIM, Orphanet, SwissProt, TCGA, GenBank, EMBL, InterPro, Reactome, 1000 Genomes, European Nucleotide Archive, Sequence Read Archive, United Kingdom Biobank, and TOPMed.

Database	Description
Ensembl	Ensembl genome browser, provides genomics sequence variation, annotates genes, and computes multiple alignments
GENCODE	Generalized Coding, identification of protein-coding genes
ClinVar	NCBI ClinVar, integration of genomic variation and human health
GeneCards	Human genes integration database
Human Gene Mutation Database (HGMD)	Human Gene Mutation Database, aligning gene lesions for human-inherited diseases
Online Mendelian Inheritance in Man (OMIM)	Online Mendelian Inheritance in man, a database on human genes and genetic phenotypes and identification of gene–disease association
Orphanet	Orphanet Rare Disease Ontology (ORDO), analyzing rare diseases and gene–disease relationship
SwissProt	Protein sequence database, information about high level of annotations
The Cancer Genome Atlas (TCGA)	The Cancer Genome Atlas, analyzation of primary cancer samples that genomically sequenced and molecularly characterized
GenBank	National Center for Biotechnology Information for the primary database
The EMBL Nucleotide Sequence Database	Molecular Biology Primary Database, in-depth data collection of primary nucleotide sequences
InterPro	Protein-based secondary database, provides protein functionality analysis and information about classification of them into families, domains, and sites
Reactome	A specialized database for metabolic and biological pathways
1000 Genomes	A comprehensive catalog of human genetic variation
European Nucleotide Archive	European Nucleotide Archive, provides nucleotide sequence information and raw sequencing data
Sequence Read Archive	Sequence Read Archive, stores raw sequencing data and alignment information
United Kingdom Biobank	Biomedical database, provides profoundly genetic and health information
Trans-Omics for Precision Medicine (TOPMed)	NHLBI Trans-Omics for Precision Medicine, provides whole-genome sequencing (WGS) and other omics data

Despite the plethora of gene–disease databases available to researchers and the healthcare industry, there are quite a few shortcomings within the databases (Huang et al., 2018). The primary issue stems from the fact that there is no standardization within these databases, as no singular database contains all the data on the currently available genome, diseases, and drugs in the market (INSERM, 1997; Stenson et al., 2012; Amberger et al., 2015; Consortium, 2015; Aken et al., 2016; Cardon and Harris, 2016; Landrum et al., 2016; Stelzer et al., 2016; Babbi et al., 2017; Huang et al., 2018; Frankish et al., 2019). In addition, these databases are often out-of-date or do not provide relevant essential information regarding diseases, reducing the practicality and usability of these databases (Chen et al., 2012; Ahmed et al., 2020b). To resolve these issues, the IOS application PAS-Gen or PROMIS-APP-SUITE has been developed to provide an accessible central database for genomic and disease information, potentially accelerating medical discoveries in the future (Stenson et al., 2017). The purpose of the app is to provide researchers and employees in the healthcare industry with information about genes that could result in the development of certain diseases for educational and non-commercial uses. In addition, the user-friendly interface of the application enables accessibility to a wide variety of users and for the app to continuously receive updates if needed (Stenson et al., 2017). This application includes a total of 59,293 genes (19,989 protein-coding and 39,304 non-protein-coding), and “is composed of 98,064 gene–disease combinations reported from 809 distinct sources” (Stenson et al., 2017). These features make the PAS App comprehensive, easily accessible, and suited for the future of personalized medicine. This centralized database and application could prove to be tremendously useful in the future, boosting the abilities of healthcare researchers to understand the human genome and its implications in the development of diseases (Stenson et al., 2017).

## Pharmacogenomics

Pharmacogenomics is the analysis of how an individual’s genome (their unique genetic makeup) influences their reaction to certain medications prescribed to them (Wake et al., 2019; Karol and Yang, 2020). Protein-coding genes could influence the treatment of the drug either by breaking it down, absorbing it, or transporting the drug to desired (or undesired) locations (Hoehe and Morris-Rosendahl, 2018; Venkatachalapathy et al., 2021). Grouping people that have similar genetic variations will allow for better observation of how these groups may have a common treatment response to a given treatment (Hoehe and Morris-Rosendahl, 2018; Wake et al., 2019b; Karol and Yang, 2020; Venkatachalapathy et al., 2021). One example of pharmacogenomics is thiopurine methyltransferase testing to determine candidates for thiopurine drug therapy, which are used to treat autoimmune disorders like Crohn’s disease or rheumatoid arthritis (Wake et al., 2019).

Pharmacogenomics has a multitude of applications for both precision medicine and related fields, allowing for the improvement of drugs in all stages of the process, from production to consumption (Wake et al., 2019; Kim et al., 2021). One such application comes in the field of drug research and production, where information from pharmacogenomics practices can help assist in how best to research potential medicine for patients for certain diseases (Penny and McHale, 2005; Payami and Factor, 2014; Relling and Evans, 2015). There have been genome-wide association studies conducted to measure responses to certain drugs, which have allowed for the identification of groups of people in which a drug may be more effective than it would be in other groups (Kim et al., 2021). Pharmacogenomics also has applications in a later step of the drug manufacturing and distribution process, primarily in the production and labeling of drugs. Pharmacogenomics information (PGx) has been increasingly included in the labels of new drug approvals, with most of these being clinically actionable, allowing for better testing and utilization in clinical practices (Cheng et al., 2021; Kim et al., 2021). A third application comes in the prescription of these drugs. For instance, the dosage and selection of drugs can be optimized with data regarding the effects of nucleotide polymorphisms on drug efficacy (Chung et al., 2020; Megías-Vericat et al., 2021).

## Discussion

Clinical genomics, AI, big data, and pharmacogenomics are essential in the future development of precision medicine (Figure 1). Precision medicine has many advantages, such as providing the means for physicians and other healthcare professionals to make more accurate diagnoses, giving healthcare officials and researchers easier access to larger amounts of medical data, and leading to a greater understanding of diseases and their underlying causes (McAlister et al., 2017; Pinho, 2017; Ginsburg and Phillips, 2018; Goetz and Schork, 2018; Bilkey et al., 2019; Ahmed et al., 2020a; Ahmed, 2020; Faulkner et al., 2020; Ahmed et al., 2021a). At present, the most prominent challenge in precision medicine is identifying which approaches to implement when working with different types of medical data (Ahmed, 2020). In addition, there is no current system in place that allows for the comparison of multi-omics patient data to provide accurate predictive and personalized results. A more user-friendly interface would be required for effective implementation of precision medicine in the healthcare field. While there have been many developments in this field in recent years, there are also many ethical and logistical challenges that are need to be addressed in clinical genomics, big data, and pharmacogenomics implementation. Furthermore, the prematurity of some these approaches, such as the pharmacogenomics field, in the scope of precision medicine present a challenge in providing reliable and accurate results.

With the rapid development of many new approaches in precision medicine, the integration of two or more fields is currently being explored. Each type of approach offers a unique understanding and perspective of the data. The integration of multiple approaches allows for a more holistic and comprehensive approach to precision medicine, incorporating information from several different fields. In precision medicine, integration of these approaches provides researchers a comprehensive understanding of a given medical case, from the cause of the disease to the relevant mechanisms and interactions associated with the disease (Hasin et al., 2017). This, in turn, allows for a more accurate selection of treatment method for a given disease. One example of the integration of these approaches in precision medicine is the application of AI to genomics. Extensive developments have been made in the intersection of the field of these two fields. AI has aided in the process of combining different algorithms to make disease analysis and predictions more efficient (Xu et al., 2019). Genomic analysis using AI is mainly focused on gene expression, DNA methylation, somatic point mutation, and copy number alteration (Xu et al., 2019). By employing machine and deep learning approaches among many others, predictive models are built that can utilize genomic and other multi-omics data to speedup the process of data analysis thus, resulting in faster decision-making (Xu et al., 2019). Different integration methods of multi-omics datasets are essential when it comes to capturing the complexity of each omics approach (Picard et al., 2021). While recent advancements have been observed in the genomics and multi-omics field, more benchmark studies are needed to choose the ideal machine-learning strategy to be implemented (Picard et al., 2021; Reel et al., 2021). Multi-omics integrative models can aid in understanding the intricacies of disease abnormalities, which is not always possible with only genomic or other single-omics analysis (Reel et al., 2021).

Although big data have some advantages in medical research, such as reduction of medical error and enablement of the correct treatment approach to a disease, the need to standardize the data content and clinical definition presents a significant disadvantage (Xu et al., 2019). Despite their own advantages in precision medicine, there are also some limitations in open access clinical data and claims data in precision medicine. One significant limitation is that the health care-specific information included in the claims can be incomplete, inaccurate, or altogether missing. This originates difficulties in determining how patient-specific treatment is appropriate or effective (Stein et al., 2014). Clinical genomics is also an emerging approach in precision medicine and involves using clinical patient data to derive relationships between genes and diseases. These gene–disease relationships are typically stored in genomic databases. These databases enable the collection and analysis of genomic data, which allows for personalized treatment of the disease before significant proliferation. Similar to open access clinical data, one major limitation of such databases is that they are not standardized, meaning data cannot be easily transferred from database-to-database. This complicates the process of cross-referencing data between different databases and can prove to be an obstacle to efficient patient treatment. Pharmacogenomics is similar to clinical genomics in that it is a branch of genomics (analysis of an individual genome). However, it focuses more on the individual’s reaction to specific treatments and medications rather than the disease itself. This is essential in precision medicine, as such study provides an insight into how patients respond to specific treatments. Such correlations can be derived between a patient’s genomic makeup and their reaction to treatments. This then allows for the grouping of patients based on similar genetic variations and allow for more precise and personalized prescription of treatment. However, pharmacogenomics is still a new field of study and has not been developed enough to be reliably utilized.

The advancements in AI, healthcare, clinical genomics, and pharmacogenomics have resulted in a substantial volume of data generated. However, this large influx of data presents an issue, as no reliable or standardized means of analysis has been developed. Such data are too large to be analyzed through common visual analysis or statistical correlation methods (Álvarez-Machancoses et al., 2020; Caudai et al., 2021). The use of AI and ML techniques alleviates this issue by allowing for the efficient management of data and providing the ability to recognize patterns in complex datasets (Caudai et al., 2021). In addition, the AI and ML techniques do not require explicit programming to complete specific tasks, as they are able to independently detect and analyze patterns within the data (Caudai et al., 2021). The implementation of such techniques and methods also provides the ability to predict an attribute based on correlated data. For example, this is especially beneficial in clinical settings, in which such techniques can be implemented to predict the pharmaceutical properties of drug targets and drug candidates (König et al., 2021). Overall, the AI and ML methods are one promising solution to the rapidly increasing volume of high-throughput data (Vadapalli et al., 2022).

In this study, we hypothesize that the continued integration of pharmacogenomics, clinical genomics, AI, and healthcare will allow for increased potential in patient diagnosis and treatment. A standardized model linking and translating all these different variables needs to be implemented at the clinical level in order to further progress in the precision medicine field. The application of clinical genomics, pharmacogenomics, artificial intelligence algorithms, and big data analytics in precision medicine across different types of patient data (multi-omics) will allow for better prognosis and prediction of disease states and their mechanisms.
